# An Examination of Demographic Involvement in Minimally Invasive Glaucoma Surgery and Cataract Surgery Clinical Trials: A Systematic Review

**DOI:** 10.3390/jcm14217861

**Published:** 2025-11-05

**Authors:** Jeremy Appelbaum, Abdullah Virk, Deepkumar Patel, Karen Allison

**Affiliations:** 1 School of Medicine, New York Medical College, Valhalla, NY 10595, USA; jappelba@student.nymc.edu; 2 College of Medicine, The University of Arizona, Phoenix, AZ 85004, USA; azvirk@arizona.edu; 3 Prevention of Blindness from Glaucoma and ARMD, Floral Park, NY 11001, USA; deepkumarptl@gmail.com; 4 Flaum Eye Institute, University of Rochester, Rochester, NY 14641, USA

**Keywords:** glaucoma, primary open angle glaucoma, cataract, minimally invasive glaucoma surgery, clinical trials, diversity in clinical trials, healthcare equity

## Abstract

**Background**: Glaucoma is the leading cause of global irreversible blindness, and it disproportionately affects people of African descent, in addition to having slightly higher prevalence rates in females. Glaucoma is a group of diseases that are characterized by progressive and irreversible damage to the optic nerve, leading to eventual blindness without proper treatment. There are a number of interventions available to treat glaucoma, including MIGS, of which usage has drastically increased due to its safety and efficacy. However, with minority populations, such as people of African descent, having the highest disease burden, it remains critical to evaluate the diversity of clinical trial populations that are used in the study of glaucoma treatments. The objective of this study is to compare the representation of Black and other ethnic minorities, as well as female participants, between cataract surgery (CS), minimally invasive glaucoma surgery (MIGS), and MIGS and cataract surgery (MACS) trials. **Methods**: This analysis consisted of publicly available data on MIGS, CS, and MACS clinical trials from 2005 to 2017, using ClinicalTrials.gov as well as prevalence data sourced from the CDC. Data reporting and synthesis adhered to PRISMA guidelines. This study focuses on sex rather than gender, as this is how data was reported on ClinicalTrials.gov. The primary outcome was the participation-to-prevalence ratio (PPR) of each clinical trial. A PPR between 0.8 and 1.2 represents adequate representation, while a PPR less than 0.8 or greater than 1.2 can signify under- or over-representation, respectively. **Results**: A total of 21 trials were included in this review, comprising 3330 clinical trial participants: 7 CS trials (*N* = 570), 13 MIGS trials (*N* = 1577), and 9 MACS trials (*N* = 1183). All of the clinical trials included data on sex, while only 14 reported race data and 7 reported ethnicity data. The overall PPR of female participants was 1.00, with CS, MIGS, and MACS clinical trials having PPRs of 0.99, 1.00, and 1.00, respectively. On the other hand, the overall PPR of Black participants was 0.44, with CS, MIGS, and MACS clinical trials having PPRs of 0.27, 0.62, and 0.22, respectively. Further analysis demonstrated that the PPR of Black participants in trials sponsored by medical device companies and medical centers or universities was 0.41 and 1.25, respectively. The study was registered with Prospero CRD420251152586. **Conclusions**: Cataract surgery, MIGS, and MIGS and cataract surgery clinical trials under-represent Black individuals and appropriately represent females. Due to the disproportionate amount of Black individuals impacted by glaucoma, this lack of representation raises concerns about the applicability of the clinical trials to these populations. Understanding clinical trial disparities in the representation of minority races is a key first step toward promoting advancements in diversity and equitable healthcare. Clinical trials in the future need to make a genuine effort to include minority groups to improve the generalizability of results.

## 1. Introduction

Glaucoma is the leading cause of irreversible blindness around the world, with estimates projecting the disease to impact 111.8 million people by 2040 [1]. Glaucoma is an optic neuropathy that results in the progressive loss of retinal ganglion cells, leading to irreversible loss of vision. Numerous risk factors influence the probability of developing glaucoma, including age, race, gender, family history, and intraocular pressure, among others. Intraocular pressure (IOP) remains the only modifiable risk factor for glaucoma, leading both existing medical and surgical treatments to target lowering the IOP [2,3]. While the exact mechanisms behind glaucoma are not completely understood, it is of the opinion that increased intraocular pressure (IOP) contributes to a higher risk of developing the condition. The Ocular Hypertension Treatment Study found that by decreasing elevated IOP levels (21–32 mmHg) by 22.5% using topical medications, the likelihood of developing primary open-angle glaucoma decreased to 4.4% from 9.5% [4]. Moreover, the most common form of glaucoma, primary open-angle glaucoma, disproportionately affects people of African descent in a more aggressive way. Gender differences also play a role, with females in the United States having a slightly higher prevalence of glaucoma according to the CDC Vision and Eye Health Surveillance System glaucoma prevalence estimates [2,5]. Hence, understanding the diversity in clinical trials targeting glaucoma therapy is crucial in gaining insight into the applicability of new and existing interventions.

There are numerous surgical and medical treatments used to treat glaucoma. Topical medications with varying mechanisms of action are commonly prescribed by providers to reduce intraocular pressure. Examples include prostaglandin analogs, beta blockers, carbonic anhydrase inhibitors, and alpha adrenergic agonists; each medication varies in side effects, efficacy, and cost [2]. Surgical interventions include glaucoma drainage devices, trabeculectomy, laser procedures like selective laser trabeculoplasty, and minimally invasive glaucoma surgeries (MIGS). MIGS has significantly grown in usage due to favorable safety profiles and modest efficacy compared to more invasive, traditional glaucoma surgeries [6,7]. This rapid growth is especially concentrated in countries like the US, where MIGS utilization grew more than 400% from 2013 to 2018 [6]. MIGS are best suited for people with ocular hypertension or mild-to-moderate glaucoma, in addition to those wanting to avoid long-term medication use [8,9].

Due to the minimally invasive nature of MIGS, these surgeries can be conducted alongside more invasive ocular procedures, such as cataract surgery, to limit the patient’s time in the operating room and also fix two problems in one visit for those who also have coexisting cataracts [10]. According to the World Health Organization, cataracts are the leading cause of global blindness, with over 94 million cases [11]. However, cataracts can be treated through surgical intervention by replacing the natural lens with an artificial lens. Due to the high prevalence of cataracts around the world, cataract surgery has grown to be the most common procedure performed worldwide [12,13]. Hence, with the growing usage of MIGS, an increasing number of clinical trials are studying the effectiveness of various MIGS devices alone and alongside more invasive surgeries such as cataract surgery. As the global population continues to age, more people will be at risk of developing glaucoma and coexisting cataracts, with studies finding that glaucoma is one of the leading comorbidities for cataracts [14]. Thus, it remains essential to elucidate the subject diversity and population representation in these clinical trials in order for providers to ascertain whether a certain treatment is proven to be clinically effective in their respective patient populations.

Interestingly, the majority of patients at risk of developing progressive blindness from open-angle glaucoma are minorities, specifically those of African descent [1]. For new clinical trials that have emerging MIGS devices and are looking into the combined effectiveness of MIGS and cataract surgery, it is becoming increasingly important to uphold diverse trial populations to improve the generalizability of the results. The FDA has passed guidelines that aim to enhance the diversity of clinical trial populations through broadening eligibility criteria [15]. However, studies have revealed that Black and Hispanic populations, amongst other minorities, are severely under-represented compared to US Census data in recent clinical trials [16]. The objective of this study is to compare the representation of Black and female participants between cataract surgery (CS), minimally invasive glaucoma surgery (MIGS), and MIGS and cataract surgery (MACS) trials.

## 2. Methods

### 2.1. Study Search and Selection

This review adheres to the Preferred Items for Systematic Reviews and Meta-Analyses (PRISMA) guidelines and focuses on peer-reviewed evidence for the devices, implants, and procedures summarized in Table 1 [17]. Checklist available in Supplementary Materials.

A comprehensive search was conducted on ClinicalTrials.gov using the following search string under condition/disease: “glaucoma”, “cataract surgery”, and “phacoemulsification”. Filters were applied to only include study type as “Interventional”, study status as “completed”, and interventions as “Device” or “Procedure”. Once filtered, the remaining records were independently screened by two reviewers according to the predefined inclusion and exclusion criteria. The search and selection were completed in June 2024. The start years of the collected trial data spanned from 2003 to 2017. Only randomized controlled trials (RCTs) published in English were included. This systematic review was registered in Prospero (CRD420251152586).

This review was deemed exempt from institutional review board approval and informed consent because it collected and synthesized non-identifiable data from previously published studies.

#### 2.1.1. Inclusion Criteria

1. MIGS clinical trials, cataract surgery clinical trials, or MIGS and cataract surgery clinical trials.
2. Completed, interventional studies.
3. Publicly available studies with results.
4. Institutional review board-approved studies.
5. Demographic subgroups including sex/gender and race.

#### 2.1.2. Exclusion Criteria

1. Follow-up shorter than 6 months.
2. Glaucoma types other than POAG (e.g., NTG or ACG).
3. Lack of data on medication usage or intraocular pressure (IOP).
4. Studies still ongoing, open to accrual, or not published in English.

#### 2.1.3. Interventions

- Cataract Surgery/Phacoemulsification.
- iStent^®^.
- CyPass^®^ Micro-Stent.
- Ex-PRESS^®^.
- Hydrus^®^.
- PRESERFLO™ MicroShunt (F.K.A., InnFocus, MIDI Arrow).
- XEN^®^ Gel Stent.

Screening of trials was carried out by reviewing the exported trial information from ClinicalTrials.Gov. Each trial was then evaluated independently for eligibility by reviewing the “Conditions, Study Type, and Intervention”. The process was made according to the PRISMA flow diagram included in Section 3.

### 2.2. Data Synthesis and Analysis

Extracted data included (1) medical intervention, (2) number of participants, (3) year the study started, (4) year the study ended, (5) region in which the study was conducted, (6) financial sponsor, (7) participant sex, (8) ethnicity (if indicated), (9) race (if indicated), and (10) age (if indicated).

The geographic region of a study was defined using the United Nations classification, which includes Africa, Asia, Europe, Latin America and the Caribbean, North America, and Oceania [18]. We also added “multiregional” as a category to account for studies that encompassed more than 1 region. Race was captured using four categories: (1) White, (2) Black, (3) Asian, and (4) other; ethnicity was recorded as Hispanic/Latino, if reported. Racial subgroups included as “other” consisted of Native Hawaiian or Pacific Islander, American Indian or Alaska Native, and unreported as defined by the US Census [19].

Sponsors for these trials were medical device companies, medical centers or universities, foundations, or government agencies. These were organized as such into (1) medical center or university, (2) medical device companies, or (3) collaborators, which included more than one sponsor type working together, as some sponsors of more than one category worked together on one trial. Details on the sponsors, interventions, trial type, regions, and a detailed summary for each of the trials can be found in the Supplement, File S1, in Tables S1–S3 and Figures S1–S4.

Statistical analyses were conducted using Microsoft Excel (Microsoft Corporation, Redmond, WA, USA) and Python, v3.10.12. Demographic data from the study population was collected, and participation by sex/gender, race, and ethnicity of each trial was calculated as a percentage of total participants in the study. Trials were grouped according to the trial start year, the region in which the trial took place, and the type of study sponsor. Descriptive statistics were obtained for all collected data.

This study looked at whether minority groups were under- or over-represented in study populations. This was accomplished by reviewing the distribution of sex, race, and ethnicity amongst participants as well as the participation-to-prevalence ratio, which is described below. This study focuses on sex rather than gender, as this is how data was reported on ClinicalTrials.gov.

### 2.3. Participation-to-Prevalence Ratio

The participation-to-prevalence ratio (PPR) is a metric used to describe the representation of specific groups in a trial relative to their representation in the disease population [20]. Trial representation was calculated by dividing the number of participants of a specific race, ethnicity, or sex in the trial by the total trial enrollment. This percentage was then divided by the prevalence of the disease (POAG) in the corresponding population. Prevalence was estimated using data from the CDC Vision and Eye Health Surveillance System, which sourced Medicare and Medicaid claims between 2014 and 2019 [21]. The proportion of the study population was then calculated by dividing the prevalence within a specific subgroup relative to the sum of the prevalence across the population. The median was then taken across each individual year where data was available.

The resultant proportion of POAG in black and female populations was found to be 22.9% and 53.7%, respectively.

**Figure 1:**
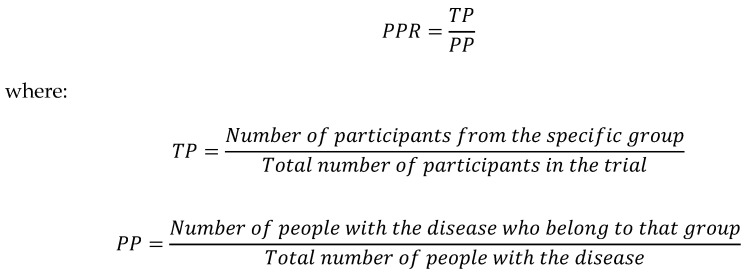
PPR formula used in analysis.

PPR is then calculated using the following formula in Figure 1:

A PPR close to 1 indicates that the subgroup proportion of the trial approximates that of the disease population. A PPR < 0.8 or >1.2 indicates that a group was under-represented or over-represented, respectively, relative to the disease population [20]. For example, consider a disease where the prevalence in the Black population is 50%. If a clinical trial in this disease were to enroll 25% Black participants, the PRR would be as follows: percentage of Black participants in the trial (25%) divided by percentage of Black patients in the disease population (50%) = 0.5, which would indicate an under-representation.

## 3. Results

### 3.1. Study Selection

**Figure 2:**
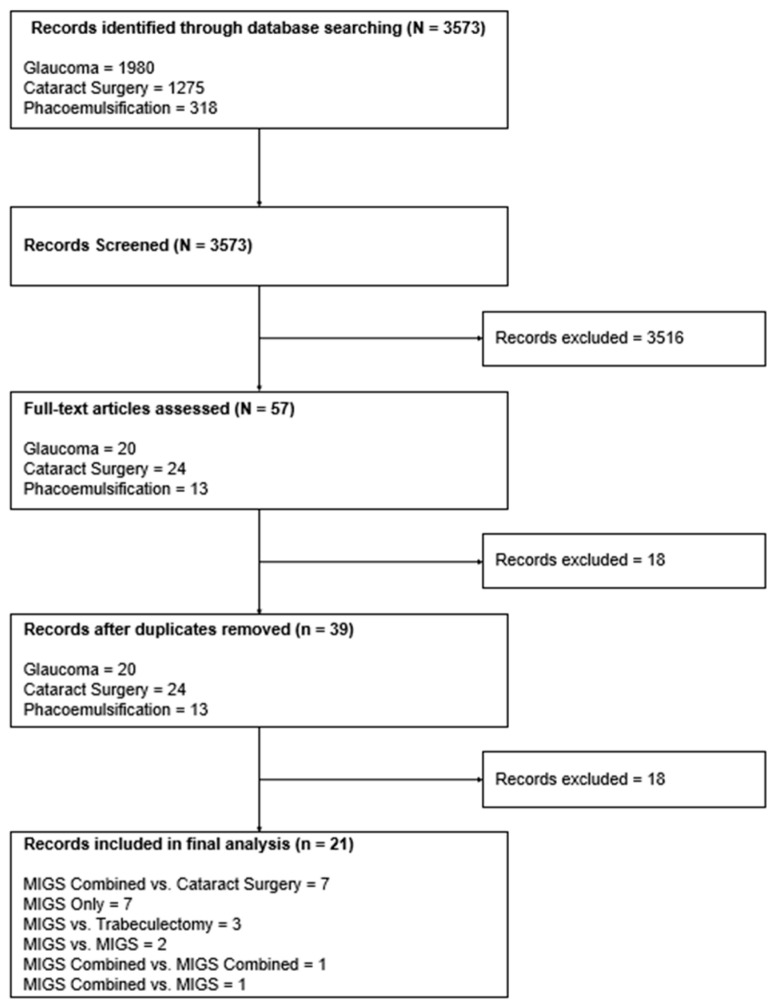
PRISMA flow diagram demonstrating number of clinical trials included and excluded.

A total of 3573 records were exported and screened from ClinicalTrials.Gov using the search strategy described above. At the end of the process, 21 clinical trials were identified and included in the analysis, which comprised 3330 participants. Seven trials compared MIGS and cataract surgery with cataract surgery alone, one trial compared MIGS and cataract surgery with MIGS and cataract surgery, one trial compared MIGS and cataract surgery with MIGS alone, two trials compared MIGS with MIGS, three trials compared MIGS with trabeculectomy, and seven trials studied MIGS alone. Figure 2 further details the included studies with a PRISMA diagram.

### 3.2. Trial Characteristics

Baseline characteristics of the included studies are reported below in Table 1, which includes the trial type, primary author, start year, ClinicalTrial.gov NCT number, trial name if available, sponsor, region, follow-up period, treatment group, and control group. Please note that two of the included studies (NCTABC and NCTX) were not found on ClinicalTrial.gov, as they were referenced separately.

All trials reported both age and sex data for trial participants; however, only 66.7% (*N* = 14) reported race, and only 33.3% (*N* = 7) reported ethnicity. For those who reported ethnicity, only two reported it accurately (i.e., distinct from race) [22,23]. The cataract surgery study by Pfeiffer, based in Europe, included no participants of Hispanic/Latino descent, while the PreserFlo MicroShunt study by Baker, based in the US and Europe, included 32 (8.1%).

### 3.3. Overall Demographics

Characteristics of all the included studies at an aggregate level are included in Table 2 below. The overall PPR for female participants was 1.0, suggesting appropriate representation. Of the trials that reported race, the black participation was 10.0% (PPR = 0.44), suggesting under-representation.

Table 3 reports the demographic characteristics for each individual clinical trial.

Female participation was 53.6% across all trials, PPR = 1.0, and was relatively similar across all intervention groups (53.3–53.8%); however, the lowest female participation by region was seen in Oceania. This was based on a single trial—a prospective, randomized trial at a single center at the Royal Victoria Eye and Ear Hospital—which had a participation rate of 32.7% (*N* = 33) [24]. Black participation was found to be 10.0% across all trials that reported race data (PPR = 0.44). By region, the lowest participation was 0.0% in Oceania (*N* = 1) and 2.9% in Europe (*N* = 2); however, it was greatest amongst MIGS cohorts (14.2%, *N* = 10) and studies sponsored by a medical center or university (28.8%, *N* = 3). Studies sponsored by medical device companies had a black participation rate of 9.4% (*N* = 10).

#### 3.3.1. Intervention Type

**Figure 3:**
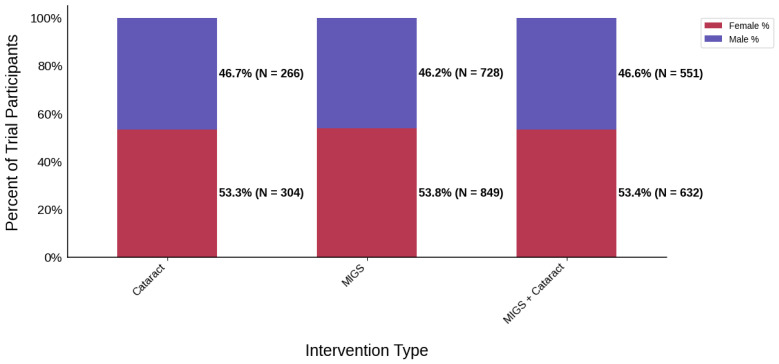
Distribution of trial participants by sex. The stacked bar chart displays the percentage of male and female participants within each intervention group.

**Figure 4:**
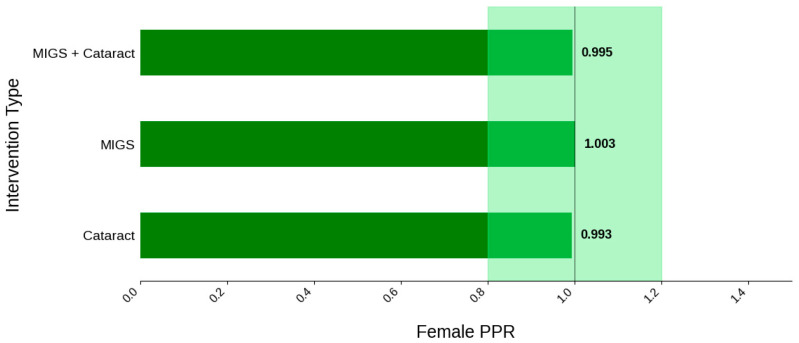
Female PPR. The Figure displays the ratio of female participation compared to the estimated prevalence by intervention group. The shaded area corresponds to a ratio of 0.8 to 1.2, which is considered an adequate representation. A ratio that is lower or higher than this range indicates an under-representation or over-representation, respectively. The green bars correspond to a PPR value that is adequately represented.

**Figure 5:**
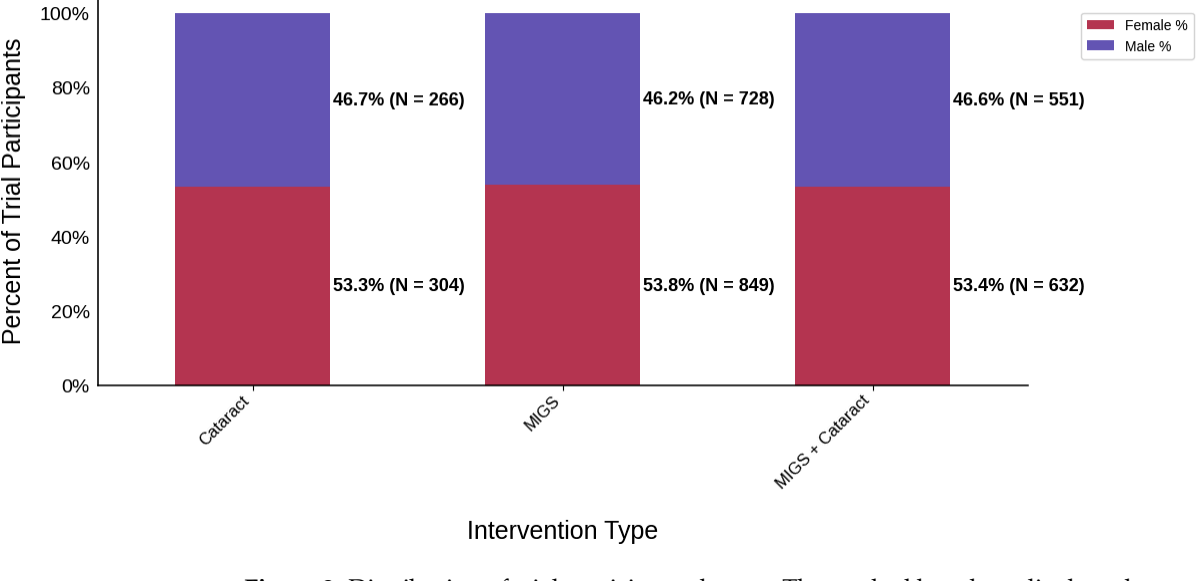
Racial distribution of trial participants. The stacked bar chart illustrates the percentage of participants by racial category within each intervention group.

**Figure 6:**
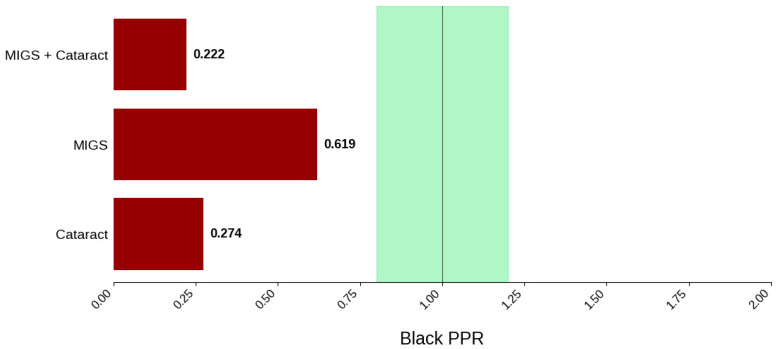
Black PPR. The Figure displays the ratio of Black participation compared to the estimated prevalence by intervention group. The shaded area corresponds to a ratio of 0.8 to 1.2, which is considered adequate representation. A ratio that is lower or higher than this range indicates an under-representation or over-representation, respectively. A red bar corresponds to a PPR value that is under or over-represented.

**Figure 7:**
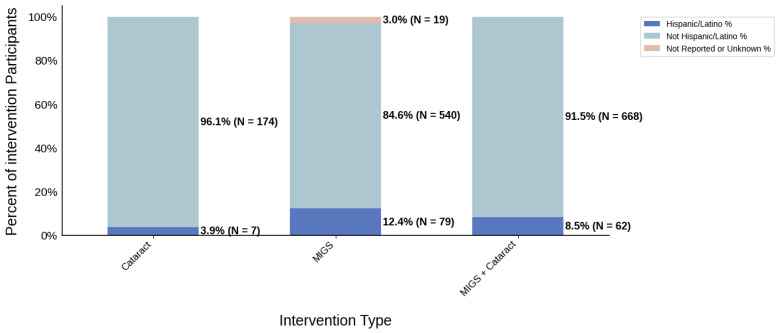
Ethnic distribution of participants. The stacked bar chart shows the percentage of participants by each ethnic category by intervention type.

Figure 3 displays the distribution of participants by sex, and Figure 4 highlights the female PPR by intervention type. In the cataract surgery group, 53.3% (*N* = 304) of participants were female, 53.8% (*N* = 849) in the MIGS group, and 53.4% (*N* = 632) in the MACS group. These distributions indicate a similar ratio across all cohorts. PPR was also found to be similar across all cohorts, 0.993 to 1.003, suggesting adequate representation. Figure 5 presents the distribution of participants by race, and Figure 6 shows the Black PPR by intervention type. The cataract surgery group had a black participation rate of 6.3% (*N* = 26), 14.2% (*N* = 197) in the MIGS group, and 5.1% (*N* = 44) in the MIGS and cataract surgery group. PPR indicates significant under-representation of Black participants across all groups. Figure 7 exhibits the ethnic distribution of participants. In the cataract surgery group, 3.9% (*N* = 7) of participants were Hispanic/Latino, 12.4% (*N* = 79) in the MIGS group, and 8.5% (*N* = 62) MIGS and cataract surgery group. This distribution indicates a higher proportion of Hispanic/Latino participants in the MIGS group compared to the other two groups. Table 4 summarizes the trial participation rate by each subgroup and intervention type, as well as the female and black PPR.

#### 3.3.2. Trial Type

**Figure 8:**
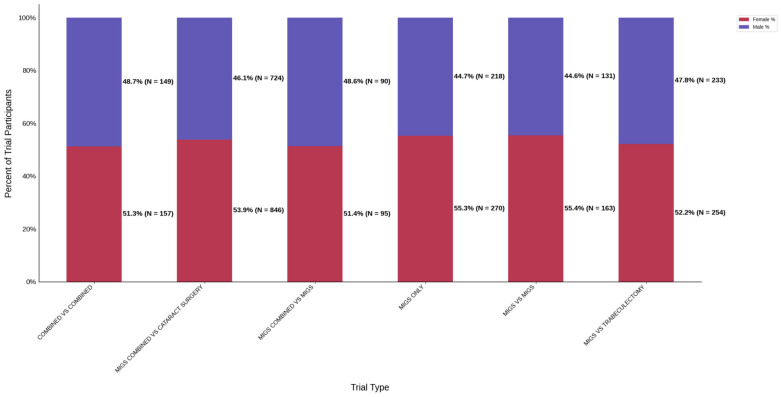
Distribution of trial participants by sex. The stacked bar chart displays the percentage of male and female participants within each trial group.

**Figure 9:**
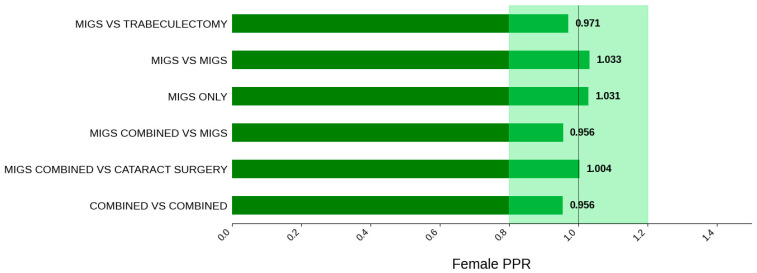
Female PPR. The Figure displays the ratio of female participation compared to the estimated prevalence by intervention group. The shaded area corresponds to a ratio of 0.8 to 1.2, which is considered adequate representation. A ratio that is lower or higher than this range indicates an under-representation or over-representation, respectively. The green bars correspond to a PPR value that is adequately represented.

**Figure 10:**
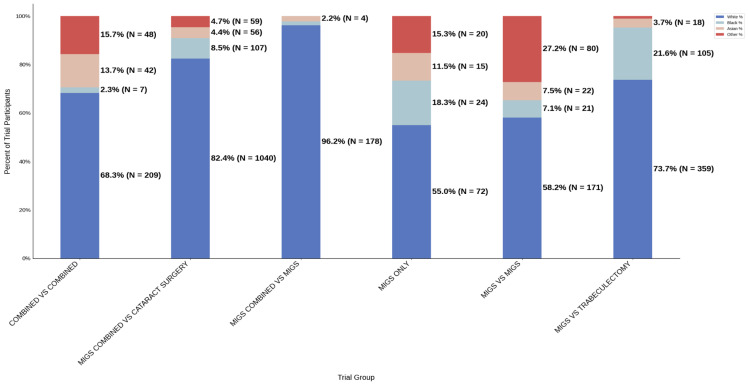
Racial distribution of trial participants. The stacked bar chart illustrates the percentage of participants by racial category for each trial group.

**Figure 11:**
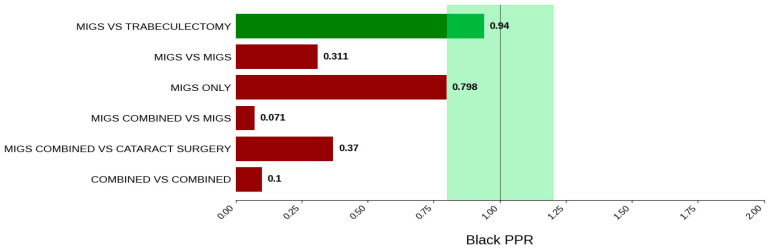
Black PPR. The Figure displays the ratio of Black participation compared to the estimated prevalence by intervention group. The shaded area corresponds to a ratio of 0.8 to 1.2, which is considered adequate representation. A ratio that is lower or higher than this range indicates an under-representation or over-representation, respectively. The green bars correspond to a PPR value that is adequately represented and a red bar corresponds to a PPR value that is under or over-represented.

**Figure 12:**
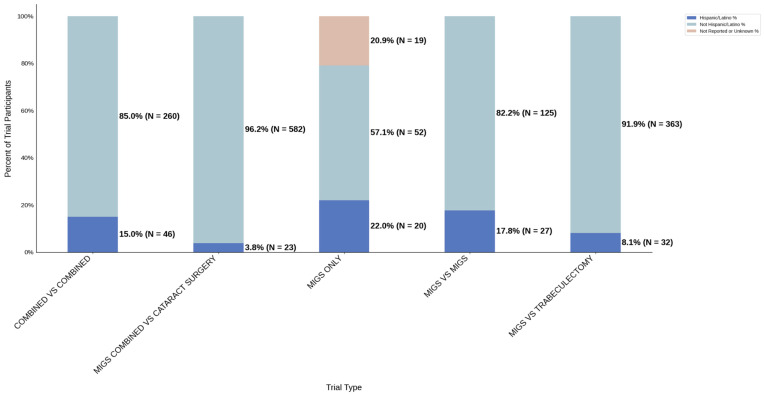
Ethnic distribution of participants. The stacked bar chart shows the percentage of participants by each ethnic category within each trial group.

Figure 8 displays the distribution of participants by sex, and Figure 9 highlights the female PPR by trial type. The female participation rate was relatively consistent across all trial types: 51.3% (combined vs. combined) to 55.4% (MIGS vs. MIGS). PPR was also found to be similar across all cohorts, 0.956 to 1.033, suggesting adequate representation. Figure 10 presents the distribution of participants by race, and Figure 11 shows the Black PPR by trial type. Black participation was greatest in the MIGS vs. trabeculectomy group at 73.7% (*N* = 359), while it was the lowest in the combined vs. MIGS group at 1.3% (*N* = 3). It is notable that the rate was 18.3% (*N* = 24) in the MIGS only group and 2.3% (*N* = 7) in the combined vs. combined group. The racial distribution varied among the groups, with the MIGS vs. MIGS group having the greatest diversity. Only the MIGS vs. trabeculectomy group had adequate representation based on PPR, with MIGS only just under the 0.8 threshold. The remaining groups had under-representation. Figure 12 exhibits the ethnic distribution of participants. The participation of Hispanic/Latinos was greater than 10% in the MIGS only, MIGS vs. MIGS, and combined vs. combined groups: 22.0%, 17.8%, and 15.0%, respectively. The lowest participation was seen in the combined vs. cataract surgery group at 3.8% (*N* = 23). Table 5 summarizes trial participation rate by each subgroup and trial type, as well as the female and black PPR.

#### 3.3.3. Region

**Figure 13:**
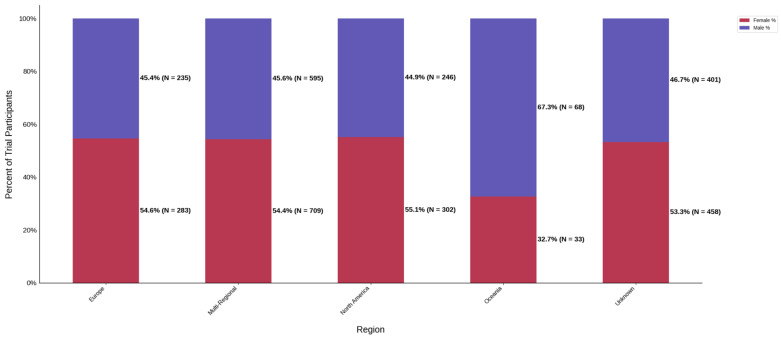
Distribution of trial participants by sex. The stacked bar chart displays the percentage of male and female participants within each region.

**Figure 14:**
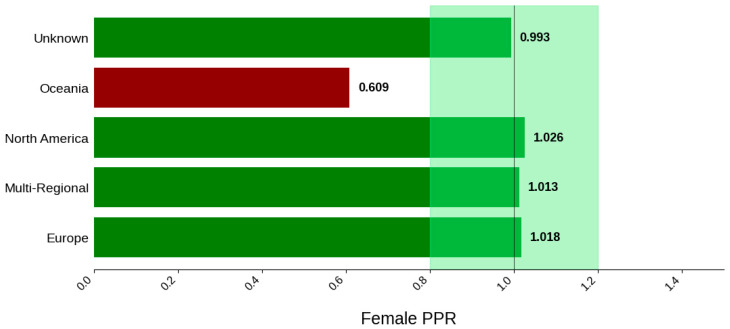
Female PPR. The Figure displays the ratio of female participation compared to the estimated prevalence by intervention group. The shaded area corresponds to a ratio of 0.8 to 1.2, which is considered adequate representation. A ratio that is lower or higher than this range indicates an under-representation or over-representation, respectively. The green bars correspond to a PPR value that is adequately represented and a red bar corresponds to a PPR value that is under or over-represented.

**Figure 15:**
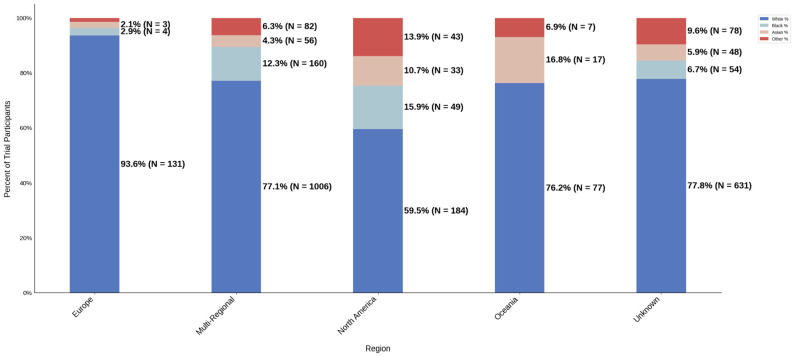
Racial distribution of trial participants. The stacked bar chart illustrates the percentage of participants by racial category within each region.

**Figure 16:**
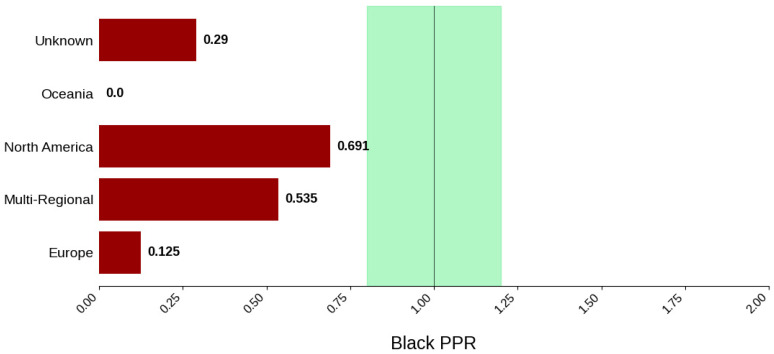
Black PPR. The Figure displays the ratio of Black participation compared to the estimated prevalence by intervention group. The shaded area corresponds to a ratio of 0.8 to 1.2, which is considered adequate representation. A ratio that is lower or higher than this range indicates an under-representation or over-representation, respectively. A red bar corresponds to a PPR value that is under or over-represented.

**Figure 17:**
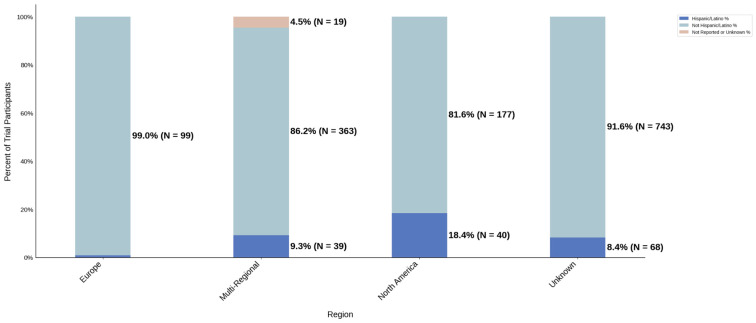
Ethnic distribution of participants. The stacked bar chart shows the percentage of participants by each ethnic category within each region.

Figure 13 displays the distribution of participants by sex, and Figure 14 highlights the female PPR by region. The sex distribution remained relatively similar across most regions, from 53.3% to 55.1%. Oceania had the lowest female participation at 32.7% (N = 33) and a PPR of 0.609, which suggests under-representation in this region. The remaining cohorts had similar female PPRs within the appropriate range. Figure 15 presents the distribution of participants by race, and Figure 16 shows the Black PPR by region. Black participation was lowest in Oceania at 0%, followed by Europe at 2.9% (N = 4). It was the highest in North America at 15.9% (N = 49); however, all groups had an under-representation of Black participants. Figure 17 exhibits the ethnic distribution of participants. North America had the highest number of reported Hispanic/Latinos at 7.3% (N = 40). Table 6 summarizes the trial participation rate by each subgroup and region as well as the female and black PPR.

#### 3.3.4. Sponsor Type

**Figure 18:**
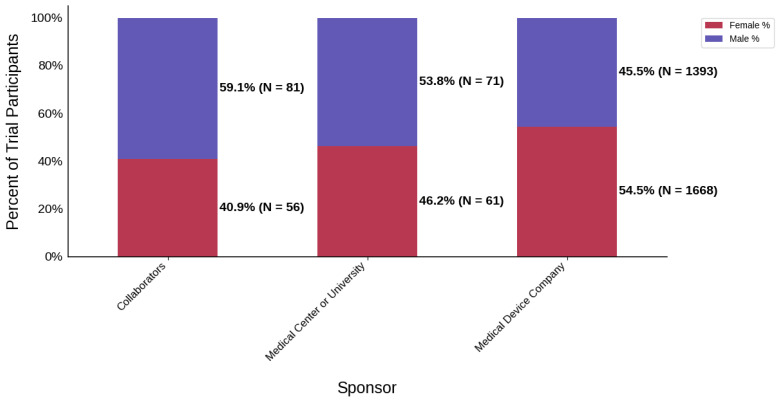
Distribution of trial participants by sex. The stacked bar chart displays the percentage of male and female participants within each sponsor group.

**Figure 19:**
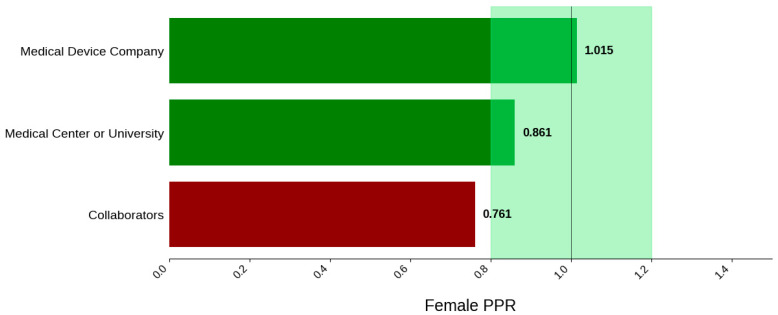
Female PPR. The Figure displays the ratio of female participation compared to the estimated prevalence by intervention group. The shaded area corresponds to a ratio of 0.8 to 1.2, which is considered adequate representation. A ratio that is lower or higher than this range indicates an under-representation or over-representation, respectively. The green bars correspond to a PPR value that is adequately represented and a red bar corresponds to a PPR value that is under or over-represented.

**Figure 20:**
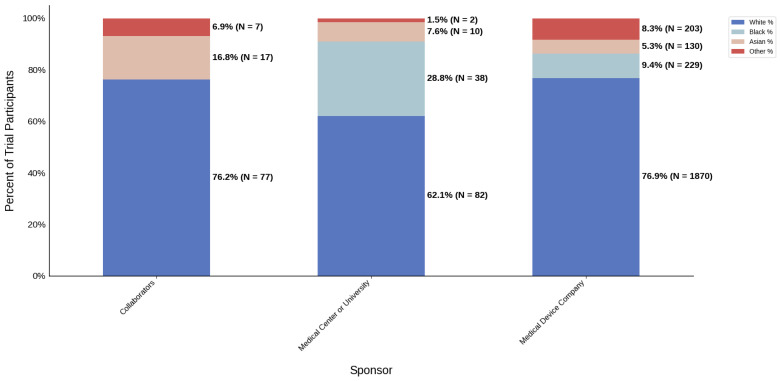
Racial distribution of trial participants. The stacked bar chart illustrates the percentage of participants by racial category within each sponsor group.

**Figure 21:**
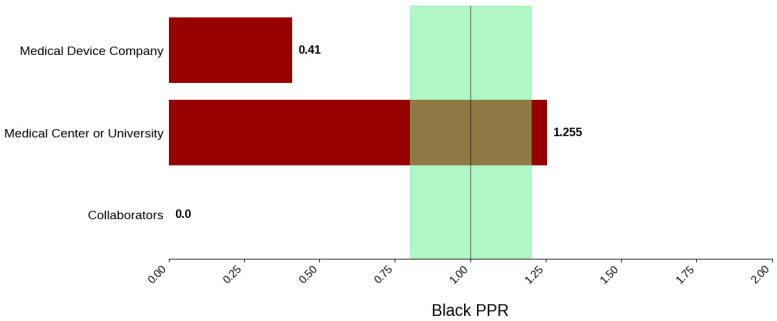
Black PPR. The Figure displays the ratio of Black participation compared to the estimated prevalence by intervention group. The shaded area corresponds to a ratio of 0.8 to 1.2, which is considered adequate representation. A ratio that is lower or higher than this range indicates an under-representation or overrepresentation, respectively. A red bar corresponds to a PPR value that is under or over-represented.

**Figure 22:**
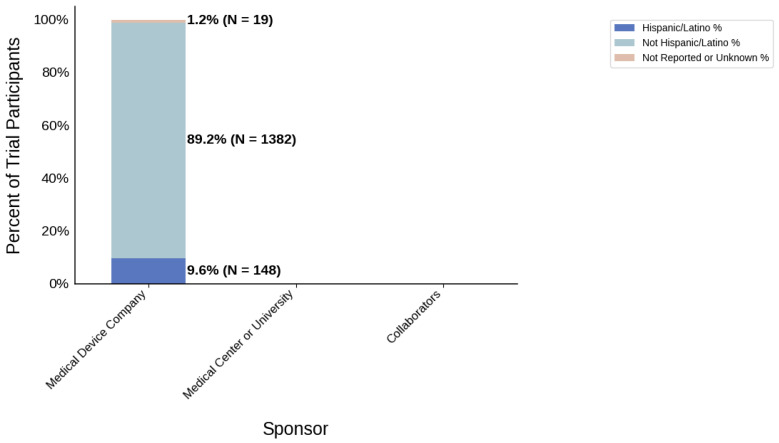
Ethnic distribution of participants. The stacked bar chart shows the percentage of participants by each ethnic category by sponsor group.

Figure 18 displays the distribution of participants by sex, and Figure 19 highlights the female PPR by sponsor type. Medical device companies had the highest female participation rate at 54.5% (*N* = 1668). Medical centers and universities had a rate of 46.2% (*N* = 61). Females were under-represented in the collaborators cohort with a PPR of 0.761. Figure 20 presents the distribution of participants by race, and Figure 21 shows the Black PPR by sponsor type. In the medical center or university group, the participation of Black individuals was 28.8% (*N* = 38), while medical device companies had a participation rate of 7.5% (*N* = 229). PPR suggests that Black participants were over-represented in the medical center or university cohort, while the other sponsor types had under-representation. Figure 22 exhibits the ethnic distribution of participants. Only medical-device-sponsored trials reported ethnicity, with Hispanic/Latinos comprising 9.6% of participants. Table 7 summarizes the trial participation rate by each subgroup and sponsor type, as well as the female and black PPR.

## 4. Discussion

We have performed a review of 21 clinical trials, consisting of 3330 participants, that studied MIGS alone, cataract surgery alone, or MIGS combined with cataract surgery. Several different types of clinical trials were included, such as MIGS combined vs. MIGS combined, MIGS combined vs. cataract surgery, MIGS combined vs. MIGS, MIGS vs. MIGS, MIGS vs. trabeculectomy, and MIGS alone. These studies primarily investigated IOP and medication reduction at 6, 12, or 24 months of follow-up. Trials utilized either a wash-out IOP baseline (unmedicated), a non-wash-out baseline (medicated), or provided both values. Additionally, we were also interested in understanding the representation of minority groups in these clinical trials by calculating PPR for female and Black participants. Overall, female and Black participants appeared adequately represented and under-represented, respectively.

### 4.1. Participation by Region, Intervention Type, Trial Type, and Sponsor Type

Region: Black participation was lowest in Oceania (0.0%) and Europe (2.9%). Female participation was also lowest in Oceania (32.7%). North America and multi-regional studies demonstrated greater racial diversity, but Black participants remained under-represented.

Intervention Type: The highest rate of Black participation was in MIGS trials (14.2%), while cataract surgery trials had lower representation (5.8%). Hispanic/Latino participation was highest in MIGS trials (12.4%) and lowest in cataract surgery trials (3.9%).

Trial Type: MIGS vs. trabeculectomy trials had the highest rate of Black participation (21.6%), while MIGS combined vs. MIGS trials had the lowest (1.6%). Female participation remained relatively balanced across trial types, with the exception of lower representation in the combined vs. combined cohort (51.3%).

Sponsor Type: Medical center or university-sponsored studies had the highest Black participation (28.8%), whereas trials led by collaborators had 0.0%. Medical device company-sponsored trials had moderate Black participation (9.4%). Further analysis demonstrated that the PPR of Black participants in trials sponsored by medical device companies and medical centers or universities was 0.41 and 1.25, respectively. A possible reason for this could be differences in recruitment strategies, geographical location, or target populations, all of which are factors influenced by external variables such as sources of funding or monetary goals. Additionally, female participation was highest in industry-sponsored trials (54.5%) and lowest in collaborator-led studies (40.9%).

### 4.2. Importance of Representation in Clinical Trials

The majority of the clinical trials in this study included primarily White participants, except for two trials: the 2006 Netland study and the 2017 Denis study. These two trials had a PPR of 2.22 and 1.51, respectively, suggesting that Black participants were over-represented. Netland studied the Ex-PRESS device compared to trabeculectomy and was sponsored by the University of Virginia. Denis studied the MINIject impact, which was conducted in India and Panama, and only included 26 participants.

Globally, the prevalence of glaucoma among individuals aged 40 to 80 years is estimated at 3.54%. Across ethnic groups, individuals of African ancestry have the highest prevalence of glaucoma (6.11%) and primary open-angle glaucoma (POAG) (5.40%), whereas Asians have the highest prevalence of primary angle-closure glaucoma (PACG) (1.20%). The odds ratio of POAG for individuals of African ancestry is 2.80 compared to those of European ancestry [1]. In this review, among studies that reported race-specific data, White participants outnumbered Black participants by approximately 7.6:1. These findings are consistent with a similar review our team conducted on MIGS clinical trials, in which the relative proportion was 4.2:1 [25]. Additionally, other studies also found that racial and ethnic minority groups are under-represented in clinical trials [26,27,28].

The racial and ethnic composition of the United States is shifting, with an increasing diversity index, which rose from 54.9% in 2010 to 61.1% in 2020. The Asian and Hispanic populations have experienced the fastest growth [29]. Given these demographic changes, there is both a clinical and ethical imperative to conduct research that reflects the diversity of the patient population. Under-representation of minority groups in clinical trials may exacerbate disparities in healthcare access, treatment utilization, and disease outcomes. Furthermore, limited diversity raises concerns regarding the generalizability of findings, as well as the safety and efficacy of surgical interventions across different racial and ethnic groups. To address these gaps, targeted initiatives are needed to promote broader representation in future clinical trials. Researchers should actively expand their study populations to ensure that minority groups are adequately included. Enhancing diversity in clinical trials is essential to developing safe, effective, and equitable treatments for all patient populations.

### 4.3. Limitations

There are several limitations to our study. First, our search was limited to the ClinicalTrials.gov database, which may introduce a geographic bias, as this registry primarily includes studies conducted in the United States. As a result, clinical trials from other regions may be under-represented, and our findings may not fully capture global trends in demographic representation. Additionally, the prevalence of POAG was estimated using data collected in the United States, which may not be representative of the populations in other regions. We also excluded ongoing trials that were still open to accrual; future analyses should explore the prevalence of minority representation in such trials.

While our analysis primarily focused on the representation of Black and female participants, we acknowledge that other racial and ethnic groups, such as Asian, Hispanic/Latino, and “Other” participants, were also reported in some studies. Future work will aim to further explore the representation and trends among these populations to provide a more comprehensive understanding of demographic inclusion in MIGS and cataract surgery clinical trials.

Another limitation was the inconsistent reporting of ethnicity. Only seven studies provided ethnicity data, suggesting that the representation of certain demographic groups may be under-reported in this review. This reflects broader disparities in clinical trial reporting, particularly regarding the Hispanic/Latino population, which is significantly affected by this disease. Furthermore, some studies categorized participants in a way that prevented individuals from identifying with multiple racial or ethnic groups. For example, certain studies only allowed participants to identify as Hispanic/Latino without specifying whether they were also Black or White. The FDA recommends that Hispanic/Latino be categorized as an ethnicity rather than a race; however, some studies classified Hispanic participants within racial categories [30]. As a result, Hispanic individuals may have been included under different racial groups in some studies, making it difficult to assess their overall representation accurately. Future research should study the collection and reporting of ethnicity data in clinical trials, as it may influence participants’ responses to interventions and have broader public health implications.

## 5. Conclusions

As glaucoma continues to grow as the world’s leading cause of irreversible blindness, new surgical procedures such as MIGS will continue to be created and applied in different settings and disease stages. MIGS has rapidly grown in popularity due to its minimally invasive nature, resulting in excellent efficacy and safety profiles, which have also enabled physicians to combine this procedure with other more invasive surgeries, such as cataract surgery. Furthermore, glaucoma continues to affect certain populations to a greater degree, with regions such as Africa facing severe disease burden and populations of African descent having higher prevalence rates. While the findings highlight appropriate representation of females and males, racial discrepancies in clinical trial representation are still present, with Black participants being severely under-represented. This under-representation raises concerns about the applicability of the clinical trial results to these populations. It is imperative that efforts to promote clinical trial diversity remain steadfast and that future clinical trials aim to improve the generalizability of the results by including under-represented populations. A noticeable number of trials in this study lacked race (33.3%) and ethnicity reporting (67.7%), proving that it is imperative for future clinical trials to have enhanced data reporting for race and ethnicity, along with the guidelines that accompany it. Future research should continue to analyze racial representation in clinical trials across the various disciplines of medicine and ophthalmology to ensure appropriate applicability of the respective results toward the general population, especially in countries with diverse populations, such as the United States. Numerous adjustments must be made concerning access and equality of care and resources to ensure that all patients receive the same standard and accessibility of care, ideally leading to better outcomes. Improved patient outcomes will result in a reduction in blindness, benefiting patients, their families, society, and the global community at large.

## Figures and Tables

**Table 1:** Baseline trial characteristics.

Trial Type	Author, Year	Sponsor	Region	Follow-Up (Months)
COMBINED vs. COMBINED	Unavailable, 2011 (NCT02024464)	Ivantis, Inc., Irvine, CA, USA	Unknown	12, 24
MIGS COMBINED vs. CATARACT SURGERY	Fan Gaskin, 2018 (NCT03106181)	Glaukos Corporation, Aliso Viejo, CA, USA; Centre for Eye Research Australia, East Melbourne, Australia	Oceania	24
MIGS COMBINED vs. CATARACT SURGERY	Fea, 2008 (NCTABC)	Glaukos Corporation; Ricerca Finalizzata della Regione Piemonte 2007	Europe	12, 15
MIGS COMBINED vs. CATARACT SURGERY	Fernandez-Barrientos, 2005 (NCT00326066)	Glaukos Corporation	Europe	6, 12
MIGS COMBINED vs. CATARACT SURGERY	Pfeiffer, 2011 (NCT01818115)	Ivantis, Inc.	Europe	12, 24
MIGS COMBINED vs. CATARACT SURGERY	Samuelson, 2005 (NCT00323284)	Glaukos Corporation	North America	12, 24
MIGS COMBINED vs. CATARACT SURGERY	Samuelson, 2012 (NCT01539239)	Ivantis, Inc.	Multi-Regional	12, 24
MIGS COMBINED vs. CATARACT SURGERY	Vold, 2009 (NCT01085357, COMPASS)	Transcend Medical, Inc., Menlo Park, CA, USA	Unknown	12, 24
MIGS COMBINED vs. MIGS	Reitsamer, 2013 (NCT02006693)	AqueSys, Inc., Aliso Viejo, CA, USA	Multi-Regional	12, 24
MIGS ONLY	Beckers, 2014 (NCT02177123)	InnFocus Inc., Three Lakes, FL, USA	Europe	12, 24
MIGS ONLY	De Jong, 2003 (NCTXYZ)	Amsterdam University Medical Center, Amsterdam, The Netherlands	Europe	12, 24
MIGS ONLY	Denis, 2017 (NCT03193736, STAR-I)	iSTAR Medical, Wavre, Belgium	Multi-Regional	6, 12, 24
MIGS ONLY	Garcia-Feijoo, 2010 (NCT01166659, DUETTE)	Transcend Medical, Inc.	Unknown	12, 24
MIGS ONLY	Grover, 2013 (NCT02036541)	AqueSys, Inc.	North America	6, 12
MIGS ONLY	Hoeh, 2009 (NCT01097174)	Transcend Medical, Inc.	Europe	6, 12
MIGS ONLY	Riss, 2011 (NCT01563237)	InnFocus Inc.	Europe	12, 24
MIGS vs. MIGS	Ahmed, 2012 (NCT02023242, COMPARE)	Ivantis, Inc.	North America	12
MIGS vs. MIGS	Unavailable, 2013 (NCT02448875, ViscoPass)	Transcend Medical, Inc.	Multi-Regional	12
MIGS vs. TRABECULECTOMY	Baker, 2015 (NCT01881425, IMS)	InnFocus Inc.	Multi-Regional	12, 24
MIGS vs. TRABECULECTOMY	Netland, 2006 (NCT00444080)	University of Virginia, Charlottesville, VA, USA	North America	6, 24
MIGS vs. TRABECULECTOMY	Wagschal, 2013 (NCT01263561)	University of Toronto, Toronto, ON, Canada	North America	12, 24

**Table 2:** Overall clinical trial demographics and PPR. Values for race and ethnicity are based on trials that reported that data.

Participants N	3330
Female N (%)	1785 (53.6)
Male N (%)	1545 (46.4)
White N (%)	2029 (76.1)
Black N (%)	267 (10.0)
Asian N (%)	157 (5.9)
Other N (%)	212 (8.0)
Hispanic/Latino N (%)	148 (9.6)
Not Hispanic/Latino N (%)	1382 (89.2)
Not Reported or Unknown N (%)	19 (1.2)
Female PPR	1
Black PPR	0.44

**Table 3:** Demographic details and PPR by clinical trial. Trials that did not report race or ethnicity statistics are left blank.

Trial Type	Author, Year	Participants N	Female N (%)	Male N (%)	White N (%)	Black N (%)	Asian N (%)	Other N (%)	Hispanic/Latino N (%)	Not Hispanic/Latino N (%)	Not Reported or Unknown N (%)	Female PPR	Black PPR
COMBINED vs. COMBINED	Unavailable, 2011 (NCT02024464)	306	157 (51.3)	149 (48.7)	209 (68.3)	7 (2.3)	42 (13.7)	48 (15.7)	46 (15.0)	260 (85.0)	0 (0.0)	0.96	0.1
MIGS COMBINED vs. CATARACT SURGERY	Fan Gaskin, 2018 (NCT03106181)	101	33 (32.7)	68 (67.3)	77 (76.2)	0 (0.0)	17 (16.8)	7 (6.9)				0.61	0
MIGS COMBINED vs. CATARACT SURGERY	Fea, 2008 (NCTABC)	36	23 (63.9)	13 (36.1)								1.19	
MIGS COMBINED vs. CATARACT SURGERY	Fernandez-Barrientos, 2005 (NCT00326066)	33	18 (54.5)	15 (45.5)								1.02	
MIGS COMBINED vs. CATARACT SURGERY	Pfeiffer, 2011 (NCT01818115)	100	51 (51.0)	49 (49.0)	97 (97.0)	0 (0.0)	1 (1.0)	2 (2.0)	1 (1.0)	99 (99.0)	0 (0.0)	0.95	0
MIGS COMBINED vs. CATARACT SURGERY	Samuelson, 2005 (NCT00323284)	239	141 (59.0)	98 (41.0)								1.1	
MIGS COMBINED vs. CATARACT SURGERY	Samuelson, 2012 (NCT01539239)	556	311 (55.9)	245 (44.1)	444 (79.9)	60 (10.8)	32 (5.8)	20 (3.6)				1.04	0.47
MIGS COMBINED vs. CATARACT SURGERY	Vold, 2009 (NCT01085357)	505	269 (53.3)	236 (46.7)	422 (83.6)	47 (9.3)	6 (1.2)	30 (5.9)	22 (4.4)	483 (95.6)	0 (0.0)	0.99	0.41
MIGS COMBINED vs. MIGS	Reitsamer, 2013 (NCT02006693)	185	95 (51.4)	90 (48.6)	178 (96.2)	3 (1.6)	4 (2.2)	0 (0.0)				0.96	0.07
MIGS ONLY	Beckers, 2014 (NCT02177123)	81	45 (55.6)	36 (44.4)								1.03	
MIGS ONLY	De Jong, 2003 (NCTXYZ)	40	21 (52.5)	19 (47.5)	34 (85.0)	4 (10.0)	2 (5.0)	0 (0.0)				0.98	0.44
MIGS ONLY	Denis, 2017 (NCT03193736)	26	12 (46.2)	14 (53.8)	0 (0.0)	9 (34.6)	10 (38.5)	7 (26.9)	7 (26.9)	0 (0.0)	19 (73.1)	0.86	1.51
MIGS ONLY	Garcia-Feijoo, 2010 (NCT01166659)	48	32 (66.7)	16 (33.3)								1.24	
MIGS ONLY	Grover, 2013 (NCT02036541)	65	35 (53.8)	30 (46.2)	38 (58.5)	11 (16.9)	3 (4.6)	13 (20.0)	13 (20.0)	52 (80.0)	0 (0.0)	1	0.74
MIGS ONLY	Hoeh, 2009 (NCT01097174)	167	99 (59.3)	68 (40.7)								1.1	
MIGS ONLY	Riss, 2011 (NCT01563237)	61	26 (42.6)	35 (57.4)								0.79	
MIGS vs. MIGS	Ahmed, 2012 (NCT02023242)	152	86 (56.6)	66 (43.4)	98 (64.5)	4 (2.6)	22 (14.5)	28 (18.4)	27 (17.8)	125 (82.2)	0 (0.0)	1.05	0.11
MIGS vs. MIGS	Unavailable, 2013 (NCT02448875)	142	77 (54.2)	65 (45.8)	73 (51.4)	17 (12.0)	0 (0.0)	52 (36.6)				1.01	0.52
MIGS vs. TRABECULECTOMY	Baker, 2015 (NCT01881425)	395	214 (54.2)	181 (45.8)	311 (78.7)	71 (18.0)	10 (2.5)	3 (0.8)	32 (8.1)	363 (91.9)	0 (0.0)	1.01	0.78
MIGS vs. TRABECULECTOMY	Netland, 2006 (NCT00444080)	59	27 (45.8)	32 (54.2)	25 (42.4)	30 (50.8)	2 (3.4)	2 (3.4)				0.85	2.22
MIGS vs. TRABECULECTOMY	Wagschal, 2013 (NCT01263561)	33	13 (39.4)	20 (60.6)	23 (69.7)	4 (12.1)	6 (18.2)	0 (0.0)				0.73	0.53

**Table 4:** Sex, racial, and ethnic distribution as well as female and Black PPR by intervention type.

Intervention Type	Cataract Surgery	MIGS	MIGS + Cataract
Female N (%)	304 (53.3)	849 (53.8)	632 (53.4)
Male N (%)	266 (46.7)	728 (46.2)	551 (46.6)
White N (%)	343 (82.9)	996 (71.8)	690 (79.9)
Black N (%)	26 (6.3)	197 (14.2)	44 (5.1)
Asian N (%)	21 (5.1)	77 (5.6)	59 (6.8)
Other N (%)	24 (5.8)	117 (8.4)	71 (8.2)
Hispanic/Latino N (%)	7 (3.9)	79 (12.4)	62 (8.5)
Not Hispanic/Latino N (%)	174 (96.1)	540 (84.6)	668 (91.5)
Not Reported or Unknown N (%)	0 (0.0)	19 (3.0)	0 (0.0)
Female PPR	0.99	1	1
Black PPR	0.27	0.62	0.22

**Table 5:** Sex, racial, and ethnic distribution as well as female and Black PPR by trial type.

Trial Type	COMBINED vs. COMBINED	COMBINED vs. CS	COMBINED vs. MIGS	MIGS ONLY	MIGS vs. MIGS	MIGS vs. TRABECULECTOMY
Female N (%)	157 (51.3)	846 (53.9)	95 (51.4)	270 (55.3)	163 (55.4)	254 (52.2)
Male N (%)	149 (48.7)	724 (46.1)	90 (48.6)	218 (44.7)	131 (44.6)	233 (47.8)
White N (%)	209 (68.3)	1040 (82.4)	178 (96.2)	72 (55.0)	171 (58.2)	359 (73.7)
Black N (%)	7 (2.3)	107 (8.5)	3 (1.6)	24 (18.3)	21 (7.1)	105 (21.6)
Asian N (%)	42 (13.7)	56 (4.4)	4 (2.2)	15 (11.5)	22 (7.5)	18 (3.7)
Other N (%)	48 (15.7)	59 (4.7)	0 (0.0)	20 (15.3)	80 (27.2)	5 (1.0)
Hispanic/Latino N (%)	46 (15.0)	23 (3.8)	N/A	20 (22.0)	27 (17.8)	32 (8.1)
Not Hispanic/Latino N (%)	260 (85.0)	582 (96.2)	N/A	52 (57.1)	125 (82.2)	363 (91.9)
Not Reported or Unknown N (%)	0 (0.0)	0 (0.0)	N/A	19 (20.9)	0 (0.0)	0 (0.0)
Female PPR	0.96	1	0.96	1.03	1.03	0.97
Black PPR	0.1	0.37	0.07	0.8	0.31	0.94

**Table 6:** Sex, racial, and ethnic distribution, as well as female and Black PPR by region.

Region	Europe	Multi-Regional	North America	Oceania	Unknown
Female N (%)	283 (54.6)	709 (54.4)	302 (55.1)	33 (32.7)	458 (53.3)
Male N (%)	235 (45.4)	595 (45.6)	246 (44.9)	68 (67.3)	401 (46.7)
White N (%)	131 (93.6)	1006 (77.1)	184 (59.5)	77 (76.2)	631 (77.8)
Black N (%)	4 (2.9)	160 (12.3)	49 (15.9)	0 (0.0)	54 (6.7)
Asian N (%)	3 (2.1)	56 (4.3)	33 (10.7)	17 (16.8)	48 (5.9)
Other N (%)	2 (1.4)	82 (6.3)	43 (13.9)	7 (6.9)	78 (9.6)
Hispanic/Latino N (%)	1 (1.0)	39 (9.3)	40 (18.4)	N/A	68 (8.4)
Not Hispanic/Latino N (%)	99 (99.0)	363 (86.2)	177 (81.6)	N/A	743 (91.6)
Not Reported or Unknown N (%)	0 (0.0)	19 (4.5)	0 (0.0)	N/A	0 (0.0)
Female PPR	1.02	1.01	1.03	0.61	0.99
Black PPR	0.12	0.53	0.69	0	0.29

**Table 7:** Sex, racial, and ethnic distribution, as well as female and Black PPR by sponsor type.

Sponsor Type	Collaborators	Medical Center or University	Medical Device Company
Female N (%)	56 (40.9)	61 (46.2)	1668 (54.5)
Male N (%)	81 (59.1)	71 (53.8)	1393 (45.5)
White N (%)	77 (76.2)	82 (62.1)	1870 (76.9)
Black N (%)	0 (0.0)	38 (28.8)	229 (9.4)
Asian N (%)	17 (16.8)	10 (7.6)	130 (5.3)
Other N (%)	7 (6.9)	2 (1.5)	203 (8.3)
Hispanic/Latino N (%)	N/A	N/A	148 (9.6)
Not Hispanic/Latino N (%)	N/A	N/A	1382 (89.2)
Not Reported or Unknown N (%)	N/A	N/A	19 (1.2)
Female PPR	0.76	0.86	1.01
Black PPR	0	1.25	0.41

## Data Availability

Not applicable.

## Supplementary Materials

The following supporting information can be downloaded at: https://www.mdpi.com/article/10.3390/jcm14217861/s1, Table S1: Interventions Included. Table S2: Types of Sponsors. Table S3: Characteristics of Clinical Trials; Figure S1: Trials Reporting Demographic Characteristics, Figure S2: Number of Trials by Region, Figure S3: Number of Trials by Sponsor Type, Figure S4: Number of Trials by Trial Type, File S1: Clinical Trial Detailed Information, File S2: PRISMA Checklist.
