# Supplementary material for: An Examination of Demographic Involvement in Minimally Invasive Glaucoma Surgery and Cataract Surgery Clinical Trials: A Systematic Review
Source: J Clin Med. 2025 Nov 5;14(21):7861. doi: 10.3390/jcm14217861 (PMC12610233; doi:10.3390/jcm14217861)
Supplement: Supplementary file 1 [file jcm-14-07861-s001.zip › jcm-3915909-supplementary.pdf]

Table S1 | Interventions Included

INTERVENTION TYPE	INTERVENTION	# OF TRIALS
**Cataract Surgery**		**7**
**MIGS**		
	CYPASS	4
	EXPRESS	3
	HYDRUS	4
	ISTENT	4
	PRESERFLO	3
	MINIJECT	1
	XEN	2
**GRAND TOTAL**		**21**

Table S2 | Types of Sponsors

SPONSOR	# OF TRIALS
**COLLABORATORS**	**2**
Glaukos Corporation &<br>Ricerca Finalizzata della Regione Piemonte 2007	1
Glaukos Corporation &<br>Centre for Eye Research Australia	1
**MEDICAL CENTERS AND UNIVERSITIES**	**3**
Amsterdam University Medical Center	1
University of Toronto	1
University of Virginia	1
**NON US MEDICAL CENTERS AND UNIVERSITIES**	**16**
AqueSys, Inc.	2
Glaukos Corporation	2
InnFocus Inc.	3
iSTAR Medical	1
Ivantis, Inc.	4
Transcend Medical, Inc.	4
**Grand Total**	**21**

Table S3 | Characteristics of Clinical Trials

CLINICAL TRIAL TYPE	# OF TRIALS
COMBINED VS COMBINED	1
MIGS COMBINED VS CATARACT SURGERY	7
MIGS COMBINED VS MIGS	1
MIGS ONLY	7
MIGS VS MIGS	2
MIGS VS TRABECULECTOMY	3
**GRAND TOTAL**	**21**

Figure S1 | Trials Reporting Demographic Characteristics

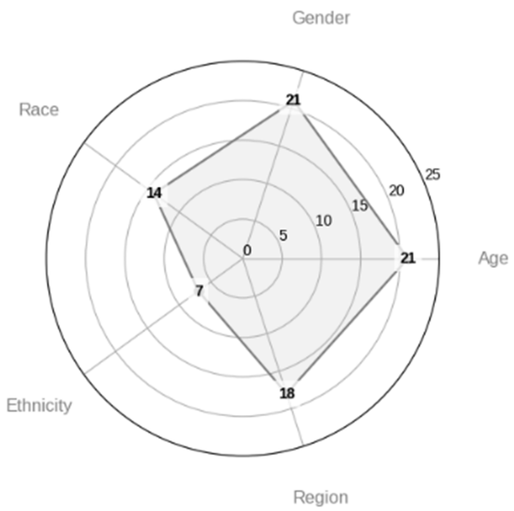

Figure S1. Radar chart showing number of trials that reported each demographic category. For example, 14 of 21 trials reported race data.

Figure S2 | Number of Trials by Region

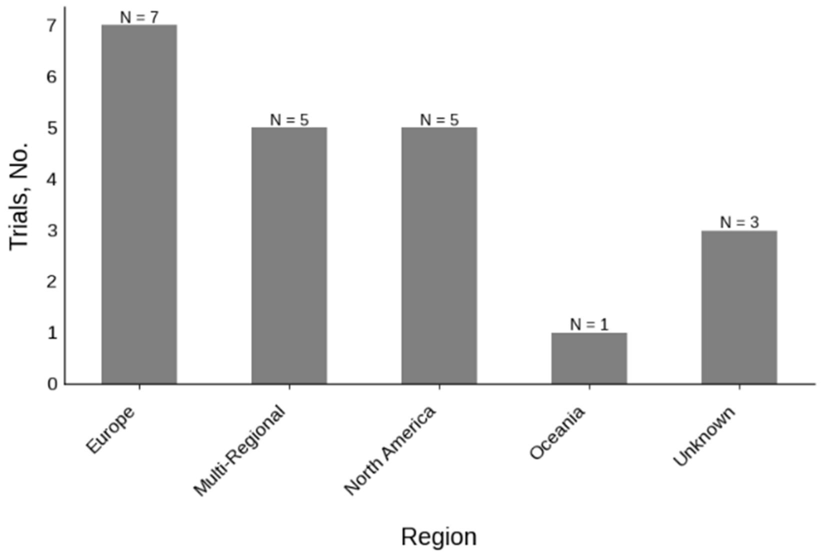

Figure S2. Bar chart showing the number of trials by region.

Figure S3 | Number of Trials by Sponsor Type

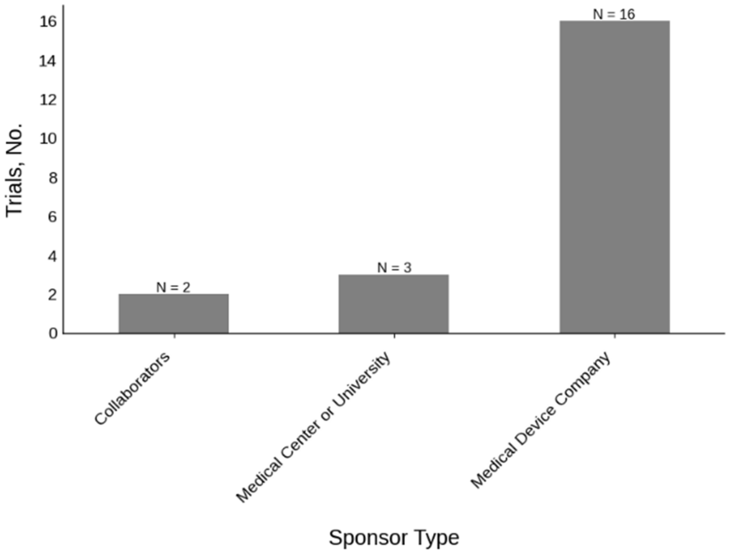

Figure S3. Bar chart showing the number of trials by sponsor type.

Figure S4 | Number of Trials by Trial Type

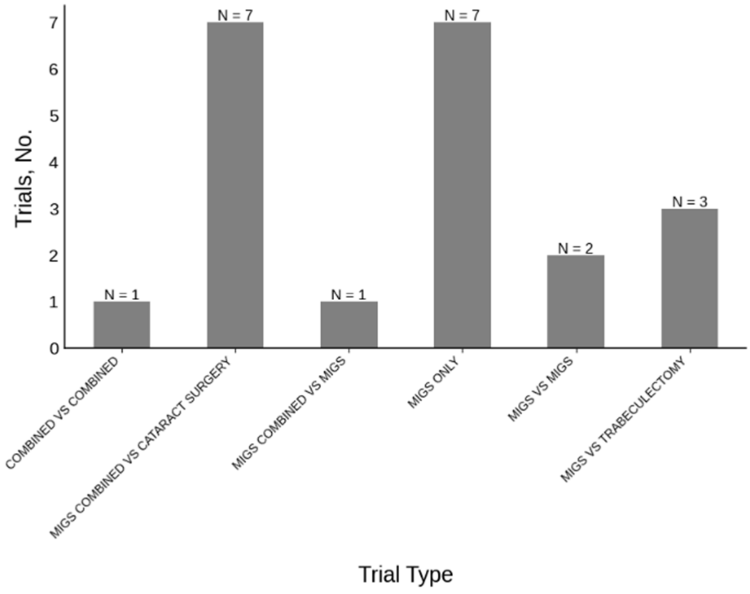

Figure A4. Bar chart showing the number of trials by trial type.

S1. Clinical Trial Detailed Information

MIGS COMBINED VS CATARACT SURGERY

Seven trials compared MIGS and cataract surgery with cataract surgery alone on a total of 1,570 patients (ISTENT, HYDRUS, CYPASS).

ISTENT

Four trials compared ISTENT and Cataract Surgery with Cataract Surgery alone on a total of 409 patients.

The 2005 Samuelson study, HORIZON, was conducted in North America and sponsored by the Glaukos Corporation.[1] They compared a combined iStent Trabecular Micro-Bypass Stent and Cataract Surgery procedure with Cataract Surgery alone on 239 patients. Female participants represented 59.0% of the study (N = 141) with a PPR of 1.10. Race and ethnicity data was not reported.

The 2008 Fea study was based in Europe and sponsored by the Ricerca Finalizzata della Regione Piemonte with devices provided by the Glaukos Corporation.[2] They compared a combined iStent Trabecular Micro-Bypass Stent and Cataract Surgery procedure with Cataract Surgery alone on 36 patients. Female participants represented 63.9% of the study (N = 23) with a PPR of 1.19. Race and ethnicity data was not reported.

The 2005 Fernandez-Barrientos study was conducted in Europe and sponsored by the Glaukos Corporation.[3] They compared a combined Two iStent Trabecular Micro-Bypass Stents and Cataract Surgery procedure with Cataract Surgery alone on 33 patients. Female participants represented 54.5% of the study (N = 18) with a PPR of 1.02. Race and ethnicity data was not reported.

The 2018 Fan Gaskin study was conducted in Australia and sponsored by the Glaukos Corporation and Centre for Eye Research Australia.[4] They compared a combined iStent Trabecular Micro-Bypass Stent and Cataract Surgery procedure with Cataract Surgery alone on

101 patients. Female participants represented 32.7% of the study (*N* = 33) with a PPR of 0.61. Only race data was reported but there were no Black participants included (PPR = 0.0).

HYDRUS

Two trials compared HYDRUS and Cataract Surgery with Cataract Surgery alone on a total of 656 patients.

The 2012 Samuelson study was Multi-Regional and sponsored by Invantis Inc.[5] They compared a combined Hydrus and Cataract Surgery procedure with Cataract Surgery alone on 556 patients. Female participants represented 55.9% of the study (*N* = 311) with a PPR of 1.04. Only race data was reported. Black participants represented 10.8% of the study (*N* = 60) with a PPR = 0.47.

The 2011 Pfeiffer study was based in Europe and sponsored by Invantis Inc.[6] They compared a combined Hydrus and Cataract Surgery procedure with Cataract Surgery alone on 100 patients. Female participants represented 51.0% of the study (*N* = 51) with a PPR of 0.95. Race and ethnicity data were both reported. There were no Black participants in the study. Hispanic/Latino participants represented 1.0% of the study (*N* = 1).

CYPASS

The 2009 Vold, COMPASS, study was sponsored by Invantis Inc.[7] They compared a combined CyPass and Cataract Surgery procedure with Cataract Surgery alone on 505 patients. Female participants represented 53.3% of the study (*N* = 269) with a PPR of 0.99. Race and ethnicity data were both reported. Black participants represented 9.3% of the study (*N* = 47) with a PPR of 0.41. Hispanic/Latino participants represented 4.4% of the study (*N* = 22).

MIGS COMBINED VS MIGS COMBINED

One trial compared MIGS and cataract surgery with MIGS and cataract surgery on a total of 306 patients (HYDRUS, ISTENT).

HYDRUS vs ISTENT

This 2011 study (author unavailable) was sponsored by Invantis Inc.[8] They compared a combined Hydrus and Cataract Surgery procedure with a combined iStent and Cataract Surgery procedure. Female participants represented 51.3% of the study (*N* = 157) with a PPR of 0.96. Race and ethnicity data were both reported. Black participants represented 2.3% of the study (*N* = 7) with a PPR of 0.10. Hispanic/Latino participants represented 15.0% of the study (*N* = 46).

MIGS COMBINED VS MIGS

One trial compared MIGS and cataract surgery with MIGS alone on a total of 185 patients (XEN).

XEN

The 2013 Reitsamer study was Multi-Regional and sponsored by AqueSys Inc.[9] They compared a combined XEN Gel Stent and Cataract Surgery procedure with XEN Gel Stent alone. Female participants represented 51.4% of the study (*N* = 95) with a PPR of 0.96. Only race data was reported. Black participants represented 1.6% of the study (*N* = 3) with a PPR of 0.07.

MIGS VS MIGS

Two trials compared MIGS alone with MIGS alone on a total of 294 patients (HYDRUS, ISTENT, CYPASS)

HYDRUS vs ISTENT

The 2012 Ahmed study was based in North America and sponsored by Ivantis Inc.[10] They compared the Hydrus Microstent with two iStents on 152 patients. Female participants represented 56.6% of the study (*N* = 86) with a PPR of 1.05. Race and ethnicity data were both reported. Black participants represented 2.6% of the study (*N* = 4) with a PPR of 0.11. Hispanic/Latino participants represented 17.8% of the study (*N* = 27).

CYPASS vs CYPASS

This 2013 ViscoPass study (author unavailable) was Multi-Regional and sponsored by Transcend Medical Inc.[11] They compared CyPass Micro-Stent and 30 µl Viscoelastic, CyPass

Micro-Stent and 60 µl Viscoelastic, CyPass Micro-Stent alone on 142 patients. Female participants represented 54.2% of the study (N = 77) with a PPR of 1.01. Only race data was reported. Black participants represented 12.0% of the study (N = 17) with a PPR of 0.52.

MIGS VS TRABECULECTOMY

Three trials compared MIGS alone with Trabeculectomy on a total of 711 patients (EXPRESS, PRESERFLO MICROSHUNT)

EXPRESS

The 2006 Netland, XVT, study was based in North America and sponsored by the University of Virginia.[12] They compared the Ex-PRESS Mini Shunt with Trabeculectomy on 120 patients. Female participants represented 45.8% of the study (N = 55) with a PPR of 0.85. Only race data was reported. Black participants represented 55.0% of the study (N = 66) with a PPR of 2.40.

The 2013 Wagschal study was based in North America and sponsored by the University of Toronto.[13] They compared the Ex-PRESS Shunt with Trabeculectomy on 64 patients. Female participants represented 35.9% of the study (N = 23) with a PPR of 0.67. Only race data was reported. Black participants represented 12.5% of the study (N = 8) with a PPR of 0.54.

PRESERFLO MICROSHUNT

The 2015 Baker, IMS, study was Multi-Regional and sponsored by InnFocus Inc.[14] They compared the InnFocus MicroShunt (N.K.A. PreserFlo MicroShunt) with Trabeculectomy on 527 patients. Female participants represented 51.8% of the study (N = 273) with a PPR of 0.96. Race and ethnicity data were both reported. Black participants represented 15.6% of the study (N = 82) with a PPR of 0.68. Hispanic/Latino participants represented 8.9% of the study (N = 47).

MIGS ONLY

Eight trials compared MIGS alone on a total of 488 patients (XEN, MINIJECT, PRESERFLO MICROSHUNT, CYPASS, EXPRESS)

XEN

The 2013 Grover study was based in North America and sponsored by AqueSys Inc.[15] They studied the XEN Gel Stent on 65 patients. Female participants represented 53.8% of the study (*N* = 35) with a PPR of 1.00. Race and ethnicity data were both reported. Black participants represented 16.9% of the study (*N* = 11) with a PPR of 0.74. Hispanic/Latino participants represented 20.0% of the study (*N* = 13).

MINIJECT

The 2017 Denis, STAR-I, study was Multi-Regional and sponsored by iSTAR Medical.[16] They studied the MINIject on 26 patients. Female participants represented 46.2% of the study (*N* = 12) with a PPR of 0.86. Race and ethnicity data were both reported. Black participants represented 34.6% of the study (*N* = 9) with a PPR of 1.51. Hispanic/Latino participants represented 26.9% of the study (*N* = 7).

PRESERFLO MICROSHUNT

Two trials studied the PRESERFLO MICROSHUNT (F.K.A MIDI Arrow, InnFocus MicroShunt) on a total of 142 patients.

The 2011 Riss study was based in Europe and sponsored by InnFocus Inc.[17] They studied the MIDI Arrow (N.K.A. PreserFlo MicroShunt) on 61 patients. Female participants represented 42.6% of the study (*N* = 26) with a PPR of 0.79. Race and ethnicity data were not reported.

The 2014 Beckers study was based in Europe and sponsored by InnFocus Inc.[18] They studied the InnFocus Microshunt (N.K.A. PreserFlo MicroShunt) on 81 patients. Female participants represented 55.6% of the study (*N* = 45) with a PPR of 1.03. Race and ethnicity data were not reported.

CYPASS

Two trials studied CYPASS on a total of 215 patients.

The 2010 Garcia-Feijoo, DUETTE, study was sponsored by Transcend Medical Inc.[19] They studied the CyPass Micro-Stent on 48 patients. Female participants represented 66.7% of the study (*N* = 32) with a PPR of 1.24. Race and ethnicity data were not reported.

The 2010 Hoeh, CyCLE, study was sponsored by Transcend Medical Inc.[20] They studied the CyPass Micro-Stent on 167 patients between those with a baseline IOP >= 21 mmHg and <21 mmHg. Female participants represented 59.3% of the study (*N* = 99) with a PPR of 1.10. Race and ethnicity data were not reported.

EXPRESS

The 2003 De Jong study was based in Europe and sponsored by Amsterdam University Medical Center.[21] They studied the Ex-PRESS Mini Shunt on 40 patients. Female participants represented 52.5% of the study (*N* = 21) with a PPR of 0.98. Only race data was reported. Black participants represented 10.0% of the study (*N* = 4) with a PPR of 0.44.

Appendix References

1. Samuelson TW, Katz LJ, Wells JM, Duh YJ, Giamporcaro JE, US iStent Study Group. Randomized evaluation of the trabecular micro-bypass stent with phacoemulsification in patients with glaucoma and cataract. *Ophthalmology*. 2011;118(3):459-467. doi:10.1016/j.ophtha.2010.07.007
2. Fea AM. Phacoemulsification versus phacoemulsification with micro-bypass stent implantation in primary open-angle glaucoma: Randomized double-masked clinical trial. *Journal of Cataract & Refractive Surgery*. 2010;36(3):407-412. doi:10.1016/j.jcrs.2009.10.031
3. Fernández-Barrientos Y, García-Feijoó J, Martínez-de-la-Casa JM, Pablo LE, Fernández-Pérez C, García Sánchez J. Fluorophotometric Study of the Effect of the Glaukos Trabecular Microbypass Stent on Aqueous Humor Dynamics. *Investigative Ophthalmology & Visual Science*. 2010;51(7):3327-3332. doi:10.1167/iovs.09-3972
4. Fan Gaskin JC, Bigirimana D, Kong GYX, et al. Prospective, Randomized Controlled Trial of Cataract Surgery vs Combined Cataract Surgery With Insertion of iStent Inject. *Ophthalmology Glaucoma*. 2024;7(4):326-334. doi:10.1016/j.ogla.2024.02.004
5. Samuelson TW, Chang DF, Marquis R, et al. A Schlemm Canal Microstent for Intraocular Pressure Reduction in Primary Open-Angle Glaucoma and Cataract: The HORIZON Study. *Ophthalmology*. 2019;126(1):29-37. doi:10.1016/j.ophtha.2018.05.012
6. Pfeiffer N, Garcia-Feijoo J, Martinez-de-la-Casa JM, et al. A Randomized Trial of a Schlemm's Canal Microstent with Phacoemulsification for Reducing Intraocular Pressure in Open-Angle Glaucoma. *Ophthalmology*. 2015;122(7):1283-1293. doi:10.1016/j.ophtha.2015.03.031
7. Vold S, Ahmed IIK, Craven ER, et al. Two-Year COMPASS Trial Results: Supraciliary Microstenting with Phacoemulsification in Patients with Open-Angle Glaucoma and Cataracts. *Ophthalmology*. 2016;123(10):2103-2112. doi:10.1016/j.ophtha.2016.06.032
8. Ivantis, Inc. *A Prospective, Multicenter, Randomized Comparison of the Hydrus Microstent to the iStent for Lowering Intraocular Pressure in Glaucoma Patients Undergoing Cataract Surgery*. clinicaltrials.gov; 2024. Accessed March 2, 2025. https://clinicaltrials.gov/study/NCT02024464
9. Reitsamer H, Sng C, Vera V, et al. Two-year results of a multicenter study of the ab interno gelatin implant in medically uncontrolled primary open-angle glaucoma. *Graefes Arch Clin Exp Ophthalmol*. 2019;257(5):983-996. doi:10.1007/s00417-019-04251-z
10. Ahmed IIK, Fea A, Au L, et al. A Prospective Randomized Trial Comparing Hydrus and iStent Microinvasive Glaucoma Surgery Implants for Standalone Treatment of Open-Angle Glaucoma: The COMPARE Study. *Ophthalmology*. 2020;127(1):52-61. doi:10.1016/j.ophtha.2019.04.034
11. Transcend Medical, Inc. *Randomized, Prospective Clinical Evaluation of the Safety and Effectiveness of Visco-Assisted CyPass® Implantation in Patients With Open Angle Glaucoma*. clinicaltrials.gov; 2019. Accessed March 1, 2025. https://clinicaltrials.gov/study/NCT02448875
12. Netland PA, Sarkisian SR, Moster MR, et al. Randomized, prospective, comparative trial of EX-PRESS glaucoma filtration device versus trabeculectomy (XVT study). *Am J Ophthalmol*. 2014;157(2):433-440.e3. doi:10.1016/j.ajo.2013.09.014
13. Wagschal LD, Trope GE, Jinapriya D, Jin YP, Buys YM. Prospective Randomized Study Comparing Ex-PRESS to Trabeculectomy: 1-Year Results. *J Glaucoma*. 2015;24(8):624-629. doi:10.1097/IJG.0000000000000029
14. Baker ND, Barnebey HS, Moster MR, et al. Ab-Externo MicroShunt versus Trabeculectomy

in Primary Open-Angle Glaucoma: One-Year Results from a 2-Year Randomized, Multicenter Study. *Ophthalmology*. 2021;128(12):1710-1721. doi:10.1016/j.ophtha.2021.05.023
15. Grover DS, Flynn WJ, Bashford KP, et al. Performance and Safety of a New Ab Interno Gelatin Stent in Refractory Glaucoma at 12 Months. *American Journal of Ophthalmology*. 2017;183:25-36. doi:10.1016/j.ajo.2017.07.023
16. Denis P, Hirneiß C, Durr GM, et al. Two-year outcomes of the MINIject drainage system for uncontrolled glaucoma from the STAR-I first-in-human trial. *Br J Ophthalmol*. 2022;106(1):65-70. doi:10.1136/bjophthalmol-2020-316888
17. Riss I. A 2-Year, Single-Center Study to Assess the Safety and Effectiveness of the MicroShunt in Primary Open-Angle Glaucoma. *Ophthalmic Research*. 2022;66(1):206-217. doi:10.1159/000526960
18. Beckers HJM, Aptel F, Webers CAB, et al. Safety and Effectiveness of the PRESERFLO® MicroShunt in Primary Open-Angle Glaucoma: Results from a 2-Year Multicenter Study. *Ophthalmology Glaucoma*. 2022;5(2):195-209. doi:10.1016/j.ogla.2021.07.008
19. Garcìa-Feijoo J, Höh H, Uzunov R, Dickerson JE. Supraciliary Microstent in Refractory Open-Angle Glaucoma: Two-Year Outcomes from the DUETTE Trial. *J Ocul Pharmacol Ther*. 2018;34(7):538-542. doi:10.1089/jop.2018.0036
20. Hoeh H, Vold SD, Ahmed IK, et al. Initial Clinical Experience With the CyPass Micro-Stent: Safety and Surgical Outcomes of a Novel Supraciliary Microstent. *J Glaucoma*. 2016;25(1):106-112. doi:10.1097/IJG.0000000000000134
21. De Jong LAMS. The Ex-PRESS glaucoma shunt versus trabeculectomy in open-angle glaucoma: a prospective randomized study. *Adv Therapy*. 2009;26(3):336-345. doi:10.1007/s12325-009-0017-6
